# Rapid Assessment of Rice Quality Traits Using Low-Cost Digital Technologies

**DOI:** 10.3390/foods11091181

**Published:** 2022-04-19

**Authors:** Aimi Aznan, Claudia Gonzalez Viejo, Alexis Pang, Sigfredo Fuentes

**Affiliations:** 1 Digital Agriculture, Food and Wine Group (DAFW), School of Agriculture and Food, Faculty of Veterinary and Agricultural Sciences, University of Melbourne, Melbourne, VIC 3010, Australia; aaznan@student.unimelb.edu.au (A.A.); cgonzalez2@unimelb.edu.au (C.G.V.); alexis.pang@unimelb.edu.au (A.P.); 2 Faculty of Chemical Engineering Technology, University Malaysia Perlis, Arau 02600, Perlis, Malaysia

**Keywords:** rice aroma, near-infrared, electronic nose, artificial neural networks, machine learning

## Abstract

Aroma and other physicochemical parameters are important attributes influencing consumer perception and acceptance of rice. However, current methods using multiple instruments and laboratory analysis make these assessments costly and time-consuming. Therefore, this study aimed to assess rice quality traits of 17 commercial rice types using a low-cost electronic nose and portable near-infrared spectrometer coupled with machine learning (ML). Specifically, artificial neural networks (ANN) were used to classify the type of rice and predict rice quality traits (aromas, color, texture, and pH of cooked rice) as targets. The ML models developed showed that the chemometrics obtained from both sensor technologies successfully classified the rice (Model 1: 98.7%; Model 2: 98.6%) and predicted the peak area of aromas obtained by gas chromatography-mass spectroscopy found in raw (Model 3: R = 0.95; Model 6: R = 0.95) and cooked rice (Model 4: R = 0.98; Model 7: R = 0.96). Furthermore, a high R = 0.98 was obtained for Model 5 to estimate the color, texture, and pH of cooked rice. The proposed method is rapid, low-cost, reliable, and may help the rice industry increase high-quality rice production and accelerate the adoption of digital technologies and artificial intelligence to support the rice value chain.

## 1. Introduction

Rice is one of the primary energy sources with a high nutritional value from fiber, minerals, proteins, vitamins, and antioxidants. It has been consumed as a staple food for more than half of the world’s population. According to Shahbandeh [1], Asian countries are the major rice consumers, China being the highest (154.9 million metric tons; MMT), followed by India (103.5 MMT), Vietnam (73.3 MMT), Bangladesh (36.7 MMT), and Indonesia (35.6 MMT). In recent years, it was observed that the growth in socioeconomic status in Asia and developing countries caused an increasing demand for rice associated with high-quality traits defined by the consumers [2,3,4]. Therefore, improving rice quality traits is vital to ensure high acceptability among consumers.

There are diverse types of rice available in the market, mainly produced to suit consumer preferences and intended cooking dishes. The diversity in rice quality may be related to the rice cultivar, cultivation practices, postharvest and milling process, storage condition, and cooking method. Aroma and texture are two important quality traits, as they may influence the market value and become the driving factors in consumer preferences for rice [5,6]. Maleki et al. [6] showed that the liking factor among regular rice consumers for cooked rice using different rice-to-water ratios was divided by their preference for fluffy or sticky texture. Besides the cooking method, amylose content in rice may also influence the rice texture, shown by the significant positive correlations between apparent amylose content and rapid visco-analyzer (RVA) pasting viscosity properties [7]. A detailed study conducted by Li et al. [8] showed that the hardness of cooked rice increased in rice cultivars that had intermediate to high amylose content. The authors suggested that it was due to the small size of amylose molecules and higher proportions of amylose branches found in the cultivars. 

The aromatic rice cultivars such as Basmati and Jasmine rice have a higher market value and preference among rice consumers than nonaromatic rice [9,10]. More than 300 volatile compounds have been found in aromatic rice cultivars [11]. Among them, 2-acetyl-1-pyrroline (2AP) is well-known as one of the key volatile aromatic compounds associated with popcorn or nutty aroma [9,12]. Besides 2-AP, Setyaningsih et al. [13] reported pentanal, hexanal, 2-pentyl-furan, 2,4-nonadienal, pyridine, 1-octen-3-ol, and (E)-2-octenal as the key markers for volatile aromatic compounds that are able to discriminate between aromatic and nonaromatic rice. Sansenya et al. [14] found that although major volatile aromatic compounds were detected in both aromatic and nonaromatic rice, higher concentration levels were observed in the aromatic rice. Likewise, the volatiles in raw and cooked rice with different degrees of milling were significantly different [15]. The latter study showed that the abundance of volatile aromatic compounds found at various concentrations forms the aroma fingerprinting, which has great potential for developing a rapid analysis method to assess rice quality traits. 

Different techniques have been used to assess rice quality traits. For example, for the aroma profile of rice, the gas chromatography-mass spectrometry (GC-MS) technique and descriptive sensory analysis have been used [12,16,17,18]. The rice texture profile is usually analyzed using the RVA and texture analyzer [7,19]. Additionally, most quality traits related to the physicochemical properties of rice are measured separately using different instruments and analysis techniques, as shown by Xia et al. [20]. However, these approaches are costly, time-consuming, and tedious. Therefore, the development of a simplified method that is robust, portable, rapid, low-cost, and appropriate to analyze different types of rice simultaneously could be very important for potential application in the rice industry. 

Electric noses (e-noses) are devices consisting of sensor arrays that are sensitive to volatile compounds, which can mimic the human olfactory system [21]. The sensors are exposed to detect the aroma profile, yielding specific volatile organic compounds (VOC) [22]. Several studies have developed or utilized commercial e-nose sensors in food and beverages, showing their potential as a promising tool for quality assessment to distinguish VOC and aroma profiles. For example, the application of e-noses has been recently reported, such as in rice [23,24], coffee [25], beer and wine [26,27], broccoli [28], apples [29], beef and pork [30,31], rapeseed [32,33], wheat bread [34], cucumbers [35], and basil [36]. On the other hand, near-infrared (NIR) spectroscopy is a rapid screening tool used to evaluate chemical and physical substances based on the interaction between the organic material and the lights ranging, for example, between 750 and 2500 nm in the electromagnetic region [37,38]. The technique offers feature information on the C-H, N-H, and O-H vibration bonds that determine the NIR spectra fingerprinting of the organic substances and VOC [39], for which the absorbance values are further correlated with the concentration of the sample [40]. This technique has been used in different studies of the food, beverage, and grain industry, including rice [41], tea [42], sugar [43], beer [27], wine [26], coffee [25], and cassava [44]. However, a limited number of studies are available on the application of e-nose and NIR spectroscopy techniques focused on developing rapid methods using low-cost and portable sensing tools, especially for the rice industry. 

Machine learning is a reliable and promising modeling tool that has been used for the prediction and automation of tasks in many industries, including medical [45], oil and gas [46], manufacturing [47], automotive [48], agriculture [49], and food [50]. Some specific applications of machine learning in the agriculture and food industries are on grapes [51], wine [52,53], beer [54,55], honey [56], and coffee [25], among others. According to previous studies, the application of sensors technology combined with machine learning has demonstrated that this technique efficiently produces high accuracy and robust prediction models [26,57,58,59]. Moreover, compared to other supervised machine learning algorithms, ANNs have been commonly used in agriculture and food studies due to their ability to predict non-linear relationships between inputs and targets [49,60]. 

This study aimed to evaluate the use of a handheld NIR spectrometer and a low-cost e-nose device designed and developed by the Digital Agriculture, Food and Wine group from The University of Melbourne (DAFW-UoM) [61] paired with machine learning modeling to classify 17 types of commercial rice (Models 1 and 2) and predict the peak area of rice aromas (Models 3, 4, 6 and 7) and other physicochemical parameters (Model 5). The study provided new insight into measuring the VOC from cooked and uncooked rice samples obtained using a low-cost e-nose for simultaneous qualitative and quantitative analysis of rice quality traits. This approach may benefit the rice industry to perform on-site quality screening, monitoring, inspection, and authentication using the reliable, non-destructive, low-cost, and rapid method.

## 2. Materials and Methods

### 2.1. Samples Preparation

In this study, 17 types of commercial rice consisting of polished and unpolished rice were purchased from local markets in Australia (Table 1). The rice was stored in its original packaging at room temperature until used for experiments. Before cooking, the rice samples were washed and strained with tap water three times. The amount of water was added to the rice according to the recommended ratio provided on the packaging with slight modification after pre-cook testing (Table 1). The rice was cooked using the steam cooking mode in a combi steamer oven (Convotherm 4 easyDial 6.10 GS, Welbilt Deutschland GmbH, Eglfing, Germany). After cooking, six cooked rice kernels were drawn from the samples to test the cooked rice. Two transparent plastic plates were used to press the rice kernels between the plates, and the rice was considered fully cooked if no white core was observed. This step is important to ensure the rice samples were thoroughly cooked before fluffing the rice with a fork and cooling it to room temperature. 

### 2.2. Near-Infrared Spectroscopy and Electronic Nose Measurements

Triplicate samples of raw rice were used to obtain the NIR absorbance value (1596–2396 nm) at every 7 to 9 nm interval using the microPHAZIR™ RX Analyzer (Thermo Fisher Scientific, Waltham, MA, USA). Calibration was conducted before the first scanning and repeated after about 10–15 scans using a standard white reference provided by the manufacturer. The measurement was obtained using a sample holder attached to the top of the scanning area to avoid environmental noise, and the samples were poured into the holder. The NIR measurements were obtained three times per replicate for each sample. The NIR absorbance values were transformed using the first derivative of the Savitsky–Golay method with polynomial order two in The Unscrambler X version 10.3 (CAMO Software, Oslo, Norway). 

A total of 60 g of raw rice samples was transferred into a 500 mL glass beaker to obtain the e-nose sensor readings (Supplementary Materials Figure S1) in triplicates. The e-nose sensor used in the study is portable and was developed by the DAFW-UoM using low-cost materials [61]. It consists of nine sensors (Henan Hanwei Electronics Co., Ltd., China) sensitive to different gases (Table 2). This e-nose has holes between each gas sensor to avoid any oversaturation from volatiles and avoid moisture accumulation. Calibration procedures were performed between each measurement for about 20 s to let the sensors reach the baseline reading before taking the new measurement. The sensors were exposed to the rice samples for one minute to obtain a stable sensor reading from the peak values. The outputs are given in volts and were analyzed using a supervised code in Matlab 2021a (Mathworks Inc., Natick, MA, USA) to detect the stable signals from the sensor readings, followed by ten equidistant subdivisions to obtain ten mean value readings per sensor [25]. The same procedure was repeated for cooked rice samples to obtain the e-nose sensor readings for the 17 rice samples. 

### 2.3. Gas Chromatography-Mass Selective Detector Analysis

A gas-chromatography with mass-selective detector 5977B (GC-MSD; Agilent Technologies, Inc., Santa Clara, CA, USA) equipped with an autosampler system PAL3 (CTC Analytics AG, Zwingen, Switzerland) was used to analyze the volatile aromatic compounds of the rice in duplicate samples. Helium was used as the gas carrier at a 1 mL min^−1^ flow rate. The raw (4 g) and cooked (3 g) rice samples were weighed and put into 20 mL screw-top vials with magnetic caps. The headspace method was used for extraction, and the samples were heated with agitation at 80 °C for 30 min to allow the rice to release the volatile aromatic compounds in the headspace of the vials, followed by the extraction using divinylbenzene–carboxen–polydimethylsiloxane (DVB–CAR–PDMS) 1.1 mm grey solid-phase microextraction (SPME) fiber [62,63] to penetrate the headspace of the vial to absorb the volatiles for 60 min under 80 °C with agitation. 

An HP-5MS (Agilent Technologies, Inc.; 30 m × 0.25 mm × 0.25 µm) column was used in the analysis; this column was selected considering other studies of rice and rice bran [64,65,66]. The SPME fiber was desorbed in the injector at 250 °C for 5 min using the splitless mode. The inlet temperature was set at 250 °C. The GC oven temperature was programmed as follows: initial temperature of 40 °C for 3 min; then increased to 100 °C at 5 °C min^−1^ and held for 3 min; and ramping to 250 °C at 10 °C min^−1^ and held for 4 min. A blank sample was placed at the start and between raw and cooked rice samples to prevent the carryover effect. The volatile aromatic compounds were matched with the National Institute of Standards and Technology (NIST; National Institute of Standards and Technology, Gaithersburg, MD, USA) library. Only identified compounds greater than 80% certainty are reported in this study. Additionally, compounds with a low relative abundance identified in less than three rice samples were omitted. 

### 2.4. Colour, pH, and Texture Measurement

The physicochemical quality of the cooked rice was measured to obtain the color, texture, and pH of samples in triplicates. The Nix Pro color sensor (illuminant: D65, observer angle: 10°; Nix Sensor Ltd., Hamilton, ON, Canada) was used to determine the L (lightness), *a* (red/green), and *b* (blue/yellow) color components of the cooked rice samples. Additionally, a pH meter, CT-6021A (Shenzhen Ke Dida Electronics Co., Ltd., Shenzhen, China), was used to determine the pH of the cooked rice samples. An amount of water was added to the sample (4 g) and mashed to form a slurry solution before measuring the pH. Ten grams of cooked rice samples were used to determine the rice texture (T) using a digital penetrometer (GY-4, China) equipped with an 8 mm diameter cylindrical probe to obtain the hardness of the rice.

### 2.5. Statistical Analysis and Machine Learning Modelling

Two matrices were developed using only the significant correlations (*p* < 0.05) between (i) the peak area of volatile aromatic compounds using GC-MS and e-nose sensor outputs for raw rice and (ii) the volatile aromatic compounds using GC-MS and e-nose, color, texture, and pH of cooked rice. 

**Figure 1:**
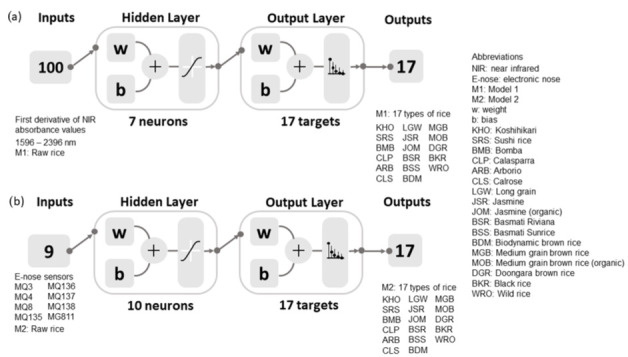
Classification models developed using (**a**) the first derivative of NIR absorbance values as inputs (Model 1) and (**b**) electronic nose sensors’ data as inputs (Model 2) to classify the 17 types of commercial rice. A description of the electronic nose sensors can be found in Table 2.

Seven machine learning models were developed by testing 17 algorithms of artificial neural network (ANN) pattern recognition in Matlab 2021a using a code developed by the DAFW-UoM [67] to find the algorithm with the best accuracy and performance with no signs of under- and overfitting, followed by a neuron trimming test (ten, seven, five, and three neurons). Pattern recognition models of ANN were developed to classify the 17 commercial rice types according to their specific types, as described in Table 1. Figure 1 shows the diagram of the models used in the study to classify the rice using as inputs the first derivative of raw rice NIR absorbance values (Model 1) and the e-nose outputs of raw rice (Model 2). The Bayesian regularization (BR) algorithm was selected for these models. It had the highest accuracy with the lowest mean squared error (MSE) using 70% training and 30% testing with random data division. 

**Figure 2:**
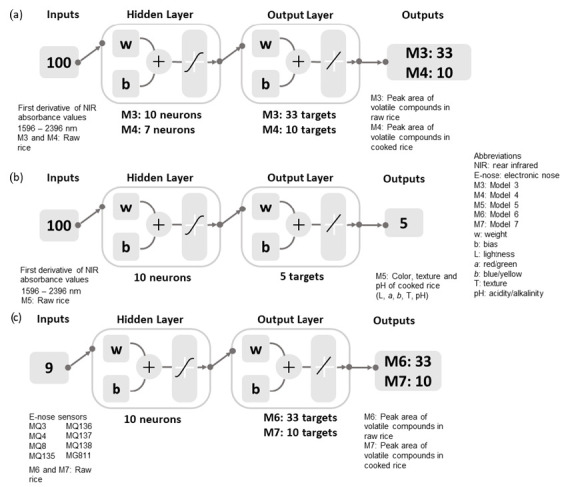
Regression models developed using the (**a**) first derivative of NIR absorbance values to estimate the aromas (Models 3 and 4) and (**b**) physicochemical quality (Model 5) of rice, and (**c**) electronic nose sensors’ data as inputs to estimate rice aromas (Models 6 and 7). A description of the electronic nose sensors can be found in Table 2.

On the other hand, ANN regression models were used to predict the aromas and other physicochemical parameters (color, texture, and pH) of the rice, as shown in Figure 2. Models 3 and 4 were developed using the first derivative of NIR spectra obtained from the raw rice as inputs to predict the abundance of volatile aromatic compounds in raw and cooked rice samples, whereas Model 5 was constructed using the raw rice NIR inputs to predict the color, texture, and pH of the cooked rice. Based on the optimization, the BR algorithm was used to develop Models 3 and 4 using random data division with 70% for training and 30% for testing, whereas Model 5 was developed using a conjugate gradient with the Powell–Beale restarts (CGB) algorithm with a random data division of 60% training, 20% validation, and 20% testing. The e-nose readings of raw rice were used as inputs to predict the peak area of volatile aromatic compounds in raw (Model 6) and cooked (Model 7) rice samples. The BR (random data division: 70% training and 30% testing) and Levenberg–Marquardt (LM; random data division: 70% training, 15% validation, and 15% testing) algorithms were used to develop Models 6 and 7, respectively.

The time required to train these ANN models depends on the characteristics of the specific computer used and the number of cores. The development of models from parameter engineering and testing of different machine learning algorithms, once all of the data are collected, can be around 2–4 h, depending on the number of models and the amount of data used. However, this process is not relevant for the deployment of the models, which can be in near-real-time. The process of the practical application of the proposed methods consists of (i) obtaining the sample, (ii) measuring with the e-nose or NIR device, and (iii) automated analysis with the machine learning model deployment. The time for this process is less than a second per sample.

## 3. Results and Discussion

**Figure 3:**
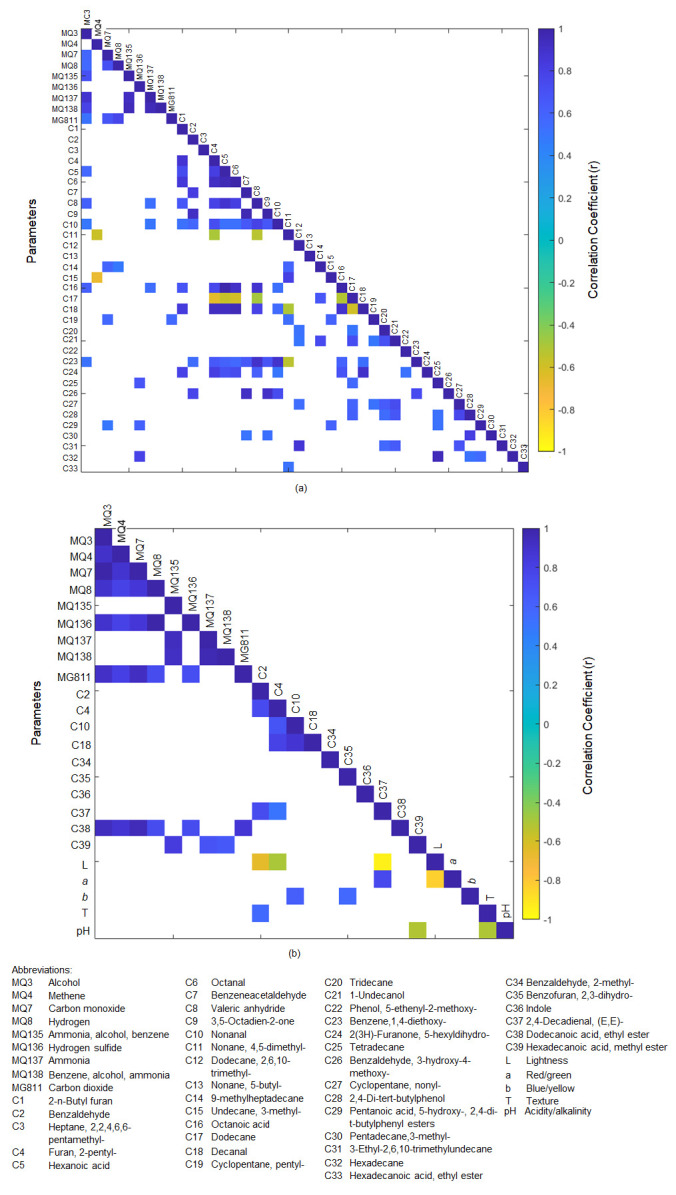
Significant correlations (*p* < 0.05) between the (**a**) electronic nose sensors’ outputs obtained from raw rice samples with volatile aromatic compounds detected in the raw rice and (**b**) electronic nose sensors’ outputs obtained from cooked rice samples with volatile aromatic compounds, color, texture, and pH of the cooked rice.

Figure 3a shows the significant correlations (*p* < 0.05) between e-nose outputs and volatile aromatic compounds found in raw rice samples. Among all e-nose sensors, MQ3 had the highest incidence of significant correlations with other variables. There were significant positive correlations between MQ3 and MQ7 (r = 0.58), MQ8 (r = 0.56), MQ135 (r = 0.71), MQ137 (r = 0.88), MQ138 (r = 0.78), MG118 (r = 0.51), and volatile aromatic compounds such as hexanoic acid (C5, r = 0.57), valeric anhydride (C8, r = 0.62), nonanal (C10, r = 0.49), octanoic acid (C16, r = 0.62), and benzene, 1,4-diethoxy (C23, r = 0.51). Additionally, it can be observed that MQ137 was positively correlated with valeric anhydride (C8, nonanal (C10, r = 0.49), and octanoic acid (C16, r = 0.62). Both nonanal and octanoic acid are associated with a waxy and fatty aroma [68]. Interestingly, positive correlations that have been reported between gas sensors and volatile aromatic compounds related to rice aging and yellowing processes, such as hexanoic acid, octanoic acid, and nonanal [18,69,70,71], were identified in the present study. This shows that the outputs obtained from the e-nose used in this study have great potential as a low-cost alternative tool for rice quality traits assessment and monitoring applications. 

Figure 3b shows the significant correlations (*p* < 0.05) between the e-nose outputs obtained from cooked rice samples with the volatile aromatic compounds, color, texture, and pH of the cooked rice samples. It was observed that the volatile compounds that belong to the esters group had a significant positive correlation with some of the e-nose sensors’ outputs. Dodecanoic acid, ethyl ester (C38) was positively correlated with MQ3 (r = 0.93), MQ4 (r = 0.87), MQ7 (r = 0.94), MQ8 (r = 0.74), MQ136 (r = 0.73), and MG811 (r = 0.87). Meanwhile, hexadecanoic acid, methyl ester (C39) was positively correlated with MQ135 (r = 0.83), MQ137 (r = 0.69), and MQ138 (r = 0.65). Dodecanoic acid, ethyl ester, is associated with sweet, waxy, floral, soapy, and clean aromas, whereas waxy and creamy are used to describe the aromas of hexadecanoic acid and methyl ester [68]. Additionally, it was observed that the lightness (L) of the cooked rice was negatively correlated with benzaldehyde (C2, r = −0.66), furan, 2-pentyl- (C4, r = −0.50), and 2,4-decadienal,(E,E) (C37, r = −0.95). This could be associated with the high abundance of the aforementioned volatile aromatic compounds detected in pigmented rice samples, such as the wild rice (WRO), which had lower L values than the other types of rice [16,72]. Even though some significant correlations were observed between e-nose outputs of cooked rice and volatile compounds detected in cooked rice samples, it will be convenient to develop a method to assess cooked rice quality traits using the raw rice samples to avoid tedious sample preparation and destructive analysis. Additionally, previous studies showed promising potential through emerging trends of using sensor technology combined with the chemometrics technique to indirectly assess cooked rice quality traits using raw rice samples [73,74,75]. 

**Figure 4:**
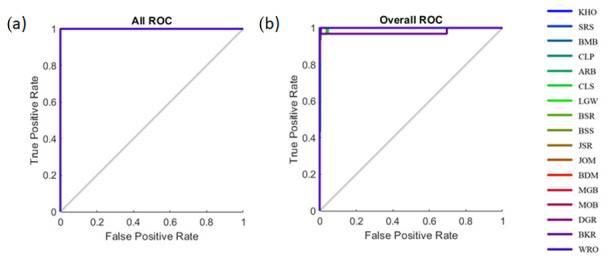
The receiver operating characteristic (ROC) curves showing the true-positive versus false-positive rates for (**a**) Model 1 and (**b**) Model 2 to classify 17 types of rice samples.

Table 3 shows the statistical results of the pattern recognition models developed to classify 17 types of commercial rice using the ANN algorithms based on the first derivative of NIR absorbances (Model 1) and e-nose readings (Model 2) as inputs. All of the classification models had high accuracy in all three stages of training, validation, and testing. The accuracies for Models 1 and 2 were high and similar (98.7% and 98.6%, respectively). Additionally, the MSE values were low, and the training had lower MSE than the testing stage, showing that the models had no sign of under- or overfitting. Figure 4 shows the overall receiver operating characteristics (ROC) curves of the classification models. From the figure, it can be observed that all of the 17 types of rice were close to the true positive rate on the vertical axis, showing that the models have high sensitivity to classify the rice samples with respect to the 17 types of rice. 

The statistical results of the regression models developed using the ANN algorithm to predict the aroma and physicochemical qualities of the rice based on the first derivative of NIR absorbance values (Model 3–Model 5) and e-nose outputs (Model 6 and Model 7) are shown in Table 4. Overall, all models had high accuracy, denoted by the correlation coefficient (R) values presented in the table. Model 3 and Model 4 developed using the BR algorithm showed high overall accuracy (Model 3: R = 0.95; Model 4: R = 0.98). Moreover, the MSE values for both models at the training stage (Model 3: MSE = 1.47 × 10^8^; Model 4: MSE = 2.22 × 10^7^) were lower than at the testing stage (Model 3: MSE = 1.23 × 10^10^; Model 4: MSE = 2.54 × 10^9^), showing the best fit models. Model 5, when predicting the color, texture, and pH of the cooked rice, showed high overall accuracy, at R = 0.96. Lower MSE values for training (MSE = 0.01) compared with the validation (MSE = 0.07) and testing (MSE = 0.08) stages and the latter two being similar confirm no signs of under- or overfitting.

The ANN regression Models 6 and 7 were developed using e-nose measurements to predict the raw and cooked rice aromas, respectively. Model 6 showed high overall accuracy (R = 0.95) and showed no overfitting signs, having a lower MSE value for training (3.37 × 10^9^) compared to validation (MSE = 3.47 × 10^9^) and testing (MSE = 5.15 × 10^9^). Additionally, Model 7 had high overall accuracy (R = 0.96) with training and testing MSE values of 5.77 × 10^8^ and 3.43 × 10^9^, respectively, showing that the MSE value for training was lower than the testing stage. 

**Figure 5:**
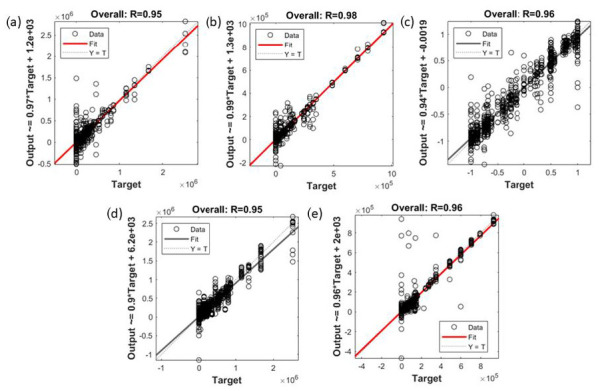
The overall correlation of the regression ANN models developed to predict rice aroma and physicochemical quality for (**a**) Model 3; inputs: first derivative of NIR absorbance; targets: raw rice aroma, (**b**) Model 4; inputs: first derivative of NIR absorbance; targets: cooked rice aroma, (**c**) Model 5; inputs: first derivative of NIR absorbance; targets: cooked rice physicochemical quality, (**d**) Model 6; inputs: e-nose outputs; targets: raw rice aroma, and (**e**) Model 7; inputs: e-nose outputs; targets: cooked rice aroma.

Figure 5 shows the overall regression models used to predict the aroma (Models 3 and 4; Models 6 and 7) and color, texture, and pH (Model 5) of the rice. The accuracy for the regression models developed using the first derivatives of NIR absorbance values (Model 3–5) and e-nose readings (Models 6 and 7) as inputs were high, with the R values of the models ranging between 0.95 and 0.98. These results are in agreement with previous studies, which obtained high accuracy predictions using ANN algorithms in food and beverages research areas, such as in coffee aroma assessment [25], mold growth prediction [76], and mulberry fruit grading [77]. It should be noted that Model 4, Model 5, and Model 7 are valid to predict the cooked rice quality traits using the standard method specified in Section 2. It is because different cooking procedures may affect the cooked rice quality, as suggested in previous studies by Maleki et al. [6], Fracassetti et al. [78], and Krongworakul et al. [79]. 

Following the present results, previous studies have shown that the chemometrics of rice obtained from low-cost sensors have been successfully applied to monitor rice quality. For example, Arjharn et al. [80] used the e-nose developed by the research team from the Agricultural Engineering Laboratory of Chiang Mai University to detect rancidity and insect infestation in brown rice during storage. The authors used the e-nose readings coupled with partial least square-discriminant analysis (PLS-DA) to classify the brown rice samples according to the normal, rancid, and infested rice groups. However, the study would have been more complete if it had included different levels of rancidity and severity of insect infestation over time to assess the sensitivity of the e-nose system. Furthermore, findings from this study supported previous studies that applied chemical and aroma fingerprinting for food authentication to detect food fraud [81,82,83].

The application of NIR absorbance values obtained in this study showed superior accuracy in predicting the physicochemical quality of the rice using the ANN modeling than those reported by Onmankhong and Sirisomboon [74]. The authors used the absorbance values measured using a Fourier transform NIR (FT-NIR) spectrometer coupled with the PSLR model to predict the hardness (coefficient of determination, R^2^ = 0.70) and toughness (R^2^ = 0.66) of cooked parboiled rice. However, the moderate accuracy of PLSR models indicated a weak predictive ability to estimate the rice quality adequately [84]. In another study, Sampaio et al. [60] compared the ability of multiple linear regression (MLP) and ANN to predict the biochemical composition and pasting parameters of rice. The authors found that the ANN (R^2^ = 0.97–0.99) presented a higher determination coefficient than the MLP (R^2^ = 0.27–0.96), showing the ability of ANN to produce a higher accuracy model. Moreover, one of the advantages of using ANN is that the model can perform multi-target prediction, while other machine learning methods are single-target [85,86]. Since NIR spectra can be used to predict multiple quality traits, the ANN model may provide a greater advantage to obtain simultaneous predictions of rice quality traits. The fact that 17 types of rice were included in this study helps generate a more general machine learning model that can be applied to classify various types of rice available in the market. This includes the types of rice from similar cultivars, subspecies (e.g., *japonica* and *indica*), and origin. 

Although previous studies had contributed to applying chemical and aroma fingerprinting in assessing rice quality traits, this study presents the feasibility of using low-cost sensors to assess rice quality traits based on qualitative and quantitative techniques. These findings suggested that the chemical fingerprinting of rice obtained using the handheld NIR spectrometer may be used to classify different types of rice and predict the aroma and physicochemical parameters of the rice. Moreover, the aroma fingerprints obtained from the e-nose sensors have great possibilities to be utilized as a reliable, low-cost alternative for rice aroma profiling and quality monitoring since the sensor can be miniaturized and installed in rice processing facilities. The method proposed in the study has some advantages in avoiding tedious sample preparation and saving time for routine inspection to assess cooked rice quality traits under similar cooking procedures. Furthermore, the proposed method may also reduce the requirement to run multiple analytical tests to assess various rice quality traits at every routine inspection (e.g., texture analyzer to determine the cooked rice texture, GC-MS analysis to determine the relative abundance of rice aroma, and other physicochemical tests). In further studies, the use of chemical fingerprinting obtained from low-cost and portable tools could be a means of a high-throughput and non-invasive approach for on-site quality inspection. This will also provide a promising solution to prevent destructive testing while obtaining the sample data. Further research should include other quality traits such as biochemical (starch, amylose, fat, and protein) data and sensory quality to assess the association between rice quality traits and consumer preferences.

## 4. Conclusions

This study set out to assess rice quality using chemical and aroma fingerprinting obtained from a handheld NIR spectrometer and low-cost e-nose sensors combined with machine learning modeling. The study has shown that the high overall accuracy of the machine learning models is robust in assessing rice quality traits. The findings suggest that the method presented in the study may allow qualitative and quantitative rice quality monitoring at a lower cost than the conventional method. The results add to the expanding application of artificial intelligence among rice producers and the government authority to monitor and assess rice quality at a reasonable price and rapidly. Further research could explore the potential application of chemical and aroma fingerprinting to predict consumer preferences concerning the various qualities of rice available in the market.

## Figures and Tables

**Table 1:** Details of commercial rice samples used in the study, including class ID, product category, type, brandabbreviation, rice-to-water ratio, and cooking time. Abbreviations: v: volume.

Class ID	Product Category	Type	Brands	Abbreviation	Rice-to-Water Ratio (*v*/*v*)	Cooking Time (min)
1	White rice (Polished rice)	Khoshihikari ^a^	SunRice	KHO	1:1 1/2	25
2		Sushi rice ^a^	SunRice	SRS	1:1 1/2	25
3		Bomba ^a^	La Perla	BMB	1:2	25
4		Calasparra ^a^	Cooperativa del Campo	CLP	1:2	25
5		Arborio ^b^	Woolworths	ARB	1:2	25
6		Calrose ^b^	SunRice	CLS	1:1 1/2	25
7		Long grain ^c^	Woolworths	LGW	1:1 1/2	25
8		Jasmine ^c^	SunRice	JSR	1:1 1/2	25
9		Jasmine-organic ^c^	Macro	JOM	1:1 1/2	25
10		Basmati ^c^	Riviana	BSR	1:1 1/3	25
11		Basmati ^c^	SunRice	BSS	1:1 1/3	25
12	Whole grain rice (Unpolished rice)	Biodynamic rice ^b^	Honest to Goodness	BDM	1:1 2/3	55
13		Medium grain ^b^	SunRice	MGB	1:1 2/3	55
14		Medium grain—organic ^b^	Macro	MOB	1:1 2/3	55
15		Doongara ^c^	SunRice	DGR	1:1 2/3	55
16		Black rice ^c^	SunRice	BKR	1:1 1/2	60
17		Wild rice—organic ^c^	Honest to Goodness	WRO	1:1 1/3	60

^a^ short grain; ^b^ medium grain; ^c^ long grain.

**Table 2:** Gas sensors integrated into the electronic nose and gases they are most sensitive.

Sensor Name	Gases
MQ3	Alcohol
MQ4	Methene
MQ7	Carbon monoxide
MQ8	Hydrogen
MQ135	Ammonia, alcohol, benzene
MQ136	Hydrogen sulfide
MQ137	Ammonia
MQ138	Benzene, alcohol, ammonia
MG811	Carbon dioxide

**Table 3:** Statistical results of Model 1 and Model 2 developed based on artificial neural network pattern recognition. Abbreviations: BR: Bayesian regularization; MSE: mean squared error; e-nose: electronic nose; NIR: near-infrared.

Algorithm	Stages	Samples	Accuracy (%)	Error (%)	Performance (MSE)
Model 1 (Inputs: NIR absorbance of raw rice; targets: 17 types of rice)					
BR 7 neurons	Training	107	100	0.0	<0.001
	Testing	46	95.7	4.3	0.003
	Overall	153	98.7	1.3	−
Model 2 (Inputs: E-nose outputs of raw rice; targets: 17 types of rice)					
BR 10 neurons	Training	357	100	0.0	<0.001
	Testing	153	95.4	4.6	0.005
	Overall	510	98.6	1.4	−

**Table 4:** Statistical results of the artificial neural network regression models developed using the first derivative of near-infrared absorbances (Model 3–Model 5) and electronic nose measurements (Model 6 and Model 7) as inputs to predict rice quality. Abbreviations: BR: Bayesian regularization; CGB: conjugate gradient with Powell–Beale restarts; LM: Levenberg–Marquardt; R: correlation coefficient; MSE: mean squared error; e-nose: electronic nose; NIR: near-infrared.

Algorithm	Stages	Samples	Observations (Samples × Targets)	R	Slope	Performance (MSE)
Model 3 (Inputs: raw rice NIR; targets: raw rice aroma)						
BR	Training	107	3531	1.00	1.00	1.47 × 10^8^
10 neurons	Testing	46	1518	0.85	0.91	1.23 × 10^10^
	Overall	153	5049	0.95	0.97	−
Model 4 (Inputs: raw rice NIR; targets: cooked rice aroma)						
BR	Training	107	1070	0.99	1.00	2.22 × 10^7^
7 neurons	Testing	46	460	0.92	0.98	2.54 × 10^9^
	Overall	153	1530	0.98	0.99	−
Model 5 (Inputs: raw rice NIR; targets: cooked rice physicochemical quality)						
	Training	91	455	0.99	0.98	0.01
CGB	Validation	31	155	0.93	0.89	0.07
10 neurons	Testing	31	155	0.90	0.88	0.08
	Overall	153	765	0.96	0.94	−
Model 6 (Inputs: raw rice e-nose; targets: raw rice aroma)						
	Training	356	11,748	0.95	0.90	3.37 × 10^9^
LM	Validation	77	2541	0.95	0.92	3.47 × 10^9^
10 neurons	Testing	77	2541	0.93	0.87	5.15 × 10^9^
	Overall	510	16,830	0.95	0.90	−
Model 7 (Inputs: raw rice e-nose; targets: cooked rice aroma)						
BR	Training	357	3570	0.98	0.97	5.77 × 10^8^
10 neurons	Testing	153	1530	0.92	0.95	3.43 × 10^9^
	Overall	510	5100	0.96	0.96	−

## Data Availability

Data and intellectual property belong to the University of Melbourne; any sharing needs to be evaluated and approved by the university.

## Supplementary Materials

The following supporting information can be downloaded at: https://www.mdpi.com/article/10.3390/foods11091181/s1, Figure S1: Set up of the electronic nose measuring a cooked rice sample.
