# Supplementary figures and images for: Rapid Assessment of Rice Quality Traits Using Low-Cost Digital Technologies
Source: Foods. 2022 Apr 19;11(9):1181. doi: 10.3390/foods11091181 (PMC9105373; doi:10.3390/foods11091181)

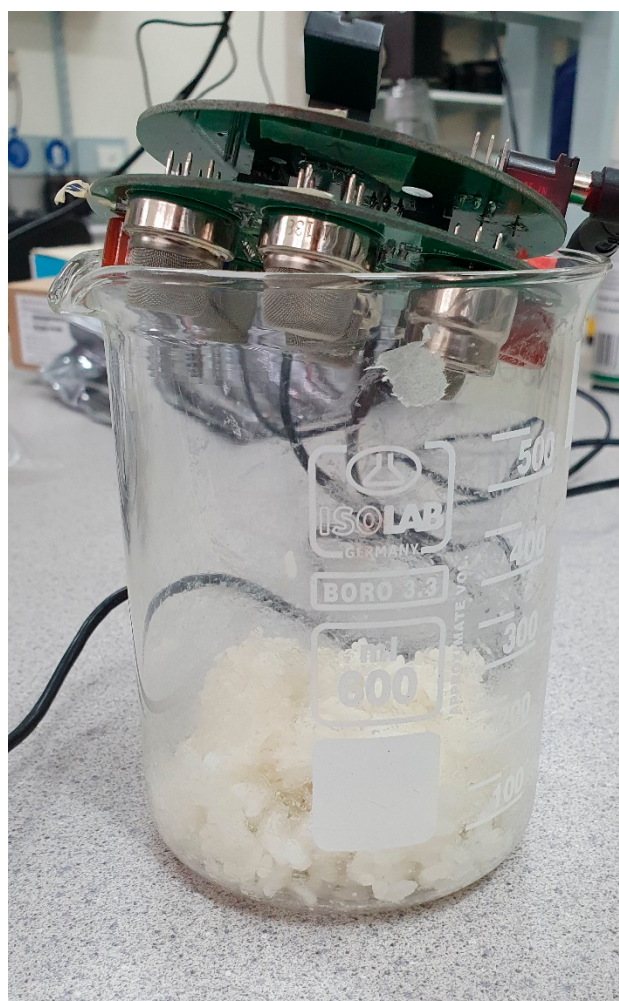

**Figure S1.** Set up of the electronic nose measuring a cooked rice sample.

Supplement: Supplementary file 1 [file foods-11-01181-s001.zip › foods-1683478-supplementary.pdf]
